# Biomimetic strategies for targeted nanoparticle delivery

**DOI:** 10.1002/btm2.10004

**Published:** 2016-05-27

**Authors:** Diana Dehaini, Ronnie H. Fang, Liangfang Zhang

**Affiliations:** ^1^ Dept. of NanoEngineering and Moores Cancer Center University of California San Diego, La Jolla CA 92093

**Keywords:** bioinspired, biomimetic nanoparticle, drug delivery, imaging, nanomedicine, targeting

## Abstract

Nanoparticle‐based drug delivery and imaging platforms have become increasingly popular over the past several decades. Among different design parameters that can affect their performance, the incorporation of targeting functionality onto nanoparticle surfaces has been a widely studied subject. Targeted formulations have the ability to improve efficacy and function by positively modulating tissue localization. Many methods exist for creating targeted nanoformulations, including the use of custom biomolecules such as antibodies or aptamers. More recently, a great amount of focus has been placed on biomimetic targeting strategies that leverage targeting interactions found directly in nature. Such strategies, which have been painstakingly selected over time by the process of evolution to maximize functionality, oftentimes enable scientists to forgo the specialized discovery processes associated with many traditional ligands and help to accelerate development of novel nanoparticle formulations. In this review, we categorize and discuss in‐depth recent works in this growing field of bioinspired research.

## 1 INTRODUCTION

The advent of nanoparticle technology has enabled the development of a wide range of novel therapeutic and diagnostic platforms. Employing nanoparticulate delivery vehicles offers many distinct advantages, including the solubilization of hydrophobic payloads, extended blood residence times, and the ability to better target a region of interest.^1^, ^2^, ^3^ One area in which nanoparticles have particularly excelled is cancer treatment, where there are numerous clinically approved nanoformulations of chemotherapeutics indicated for a variety of cancer types.^4^ At their size scale, nanoparticles can take advantage of the enhanced permeation and retention (EPR) effect, which enables them to preferentially localize to tumor sites.^5^, ^6^ First‐generation nanoparticle therapeutics generally relied on this passive targeting mechanism to improve efficacy over traditional free‐drug formulations, which often have severe systemic side effects. As nanoparticle technology has further matured, researchers have found ways to introduce specific ligands onto their surfaces. This has enabled the development of actively targeted formulations, which take advantage of very specific binding interactions with their targets to further enhance delivery. Such an active targeting strategy enables the fabrication of nanoparticle platforms for the treatment and detection of diseases beyond cancer, where the EPR effect does not exist. As the result of significant research on the subject, there are many targeted nanoformulations that are currently under clinical investigation.^7^

Conventional targeting ligands can broadly be characterized into a few different categories, including small molecules, peptides, aptamers, and antibodies. These ligands are generally incorporated onto nanoparticle surfaces by means of facile conjugation chemistries, either pre‐ or post‐synthesis.^8^, ^9^ Other strategies involve preconjugation onto an anchoring molecule followed by postinsertion via physical interactions.^10^ While most of these conventional ligands exist in certain forms within living systems,^11^ their use as agents for targeted delivery generally depends on workflows to create novel specificities not found naturally. This is done via approaches that require extensive screening. For example, new aptamers or peptides with high binding affinities to desired targets can be discovered via systematic evolution of ligands by exponential enrichment (SELEX) or phage display, respectively.^12^, ^13^ Monoclonal antibodies are generated by screening hybridomas derived from the B cells of antigen‐exposed organisms. Because, in the absence of autoimmunity, B cells that produce antibodies against most endogenous targets are usually subject to negative selection, most antibodies for clinical use further go through a humanization process.^14^, ^15^ Ultimately, these methods of targeting ligand generation enable the creation of wide‐ranging specificities against almost any target.

**Figure 1:**
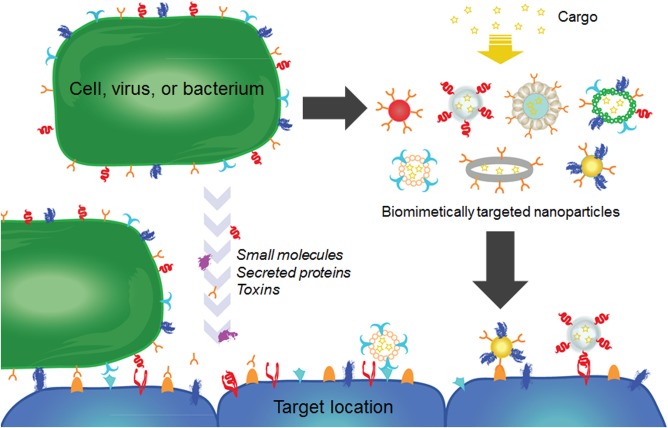
Schematic of biomimetic targeting strategies. Targeting affinities exist among cells, small molecules, proteins, and toxins. These can be directly leveraged to create targeted nanoparticle formulations for therapeutics and imaging

Despite the flexibility offered by conventional routes of targeting ligand discovery, there has more recently been a significant focus on deliberately leveraging targeting specificities found directly in nature for the creation of targeted nanodelivery systems (Figure 1). The movement toward biomimetic systems has been ushered in by an increased understanding of the different biological interactions that exist within the body.^16^, ^17^ One major advantage of such an approach is the reliance on biological mechanisms that have already been optimized by evolution over the course of millions of years. In the field of nanomedicine, one recent example has involved the enhancement of nanoparticle circulation time and the immune evasion properties.^18^ This is an area where synthetic polyethylene glycol (PEG) coatings, which present a steric barrier for decreased biological interactions, have long been the gold standard.^19^, ^20^ Biomimetic methods, rather than attempt to stealth themselves from the immune system, directly interact with it in order to convince the body that they belong to. Examples include use of red blood cell (RBC)‐derived immunomodulatory signals or even the direct use of RBC membrane as a surface coating material.^21^, ^22^, ^23^ Regarding targeting applications, there are countless receptor‐ligand, binding, and adhesion interactions that can be taken advantage of. Natural targeting mechanisms exist both within and across different species; they can be sourced from many different organisms, ranging from pathogens to mammalian cells, and vary greatly in form and function, ranging from small molecules for targeting nutrient receptors to whole cell membranes for the faithful replication of multiple cell functions. Taking advantage of the way cells naturally interact with each other and their environment opens new avenues for the development of targeted therapeutics and diagnostics.

In this review, we specifically focus on biomimetic nanoparticle platforms that employ targeting specificities found naturally in living systems. We begin our discussion with the use of individual targeting ligands such as small molecules, carbohydrate‐based structures, and peptides. Also in this category is the use of whole proteins, both secreted and membrane‐bound. Subsequently, pathogen‐derived nanostructures, both from viruses and bacteria, are covered. The final topic we will discuss involves the direct use of cellular membranes as a means to introduce both nanoparticle targeting functionality and efficient interfacing capabilities with the body's biological systems. In each section, we first introduce the targeting functionality in its natural context before describing current works that employ it. The review concludes with our thoughts on the future of biomimetic targeting.

## 2 SMALL MOLECULES

### 2.1 Folate

**Figure 2:**
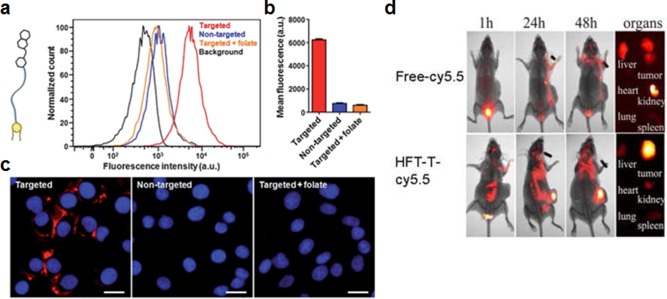
Examples of targeting strategies using small molecules. (a–c) Targeting of red blood cell membrane‐coated nanoparticles (RBC‐NPs) with folate using a membrane‐anchoring approach. (a) Schematic of a folate molecule conjugated to a lipid‐PEG tether along with a flow cytometry histogram demonstrating that targeted RBC‐NPs display increased affinity to folate receptor‐overexpressing cells. The targeting is abrogated in the presence of free folate. (b) Quantification of mean fluorescence intensities from the experiment in (a). (c) Fluorescent imaging demonstrating targeting capability of targeted RBC‐NPs. Red: dye labeled nanoparticles; blue: DAPI‐stained nuclei. Scale bar = 25 µm. (d) Heparin‐folate‐paclitaxel nanoparticles (HFT‐Ts) target folate receptors in a KB xenograft tumor model in vivo. Particles were labeled with Cy5.5 dye for in vivo fluorescence imaging. (a–c) Adapted with permission from ref. 10. Copyright 2013 by The Royal Society of Chemistry. (d) Adapted with permission from ref. 30. Copyright 2011 by American Chemical Society

Folate, or vitamin B_9_, is an example of a naturally occurring ligand that has been widely used by researchers to introduce targeting functionalization onto nanoparticles. The molecule is essential to cellular function, as it plays a major role in DNA and RNA production.^24^ On cancer cells, the overexpression of folate receptors is commonly seen, which helps to drive their aggressive phenotypes.^25^, ^26^ It has been demonstrated that, by adding folate molecules to the surface of nanoparticles, it is possible to preferentially target folate receptor‐overexpressing cancer cells.^27^, ^28^ For drug delivery, this has enabled more effective localization of nanoparticle‐carrying chemotherapeutics to tumor sites.^27^, ^28^, ^29^ RBC membrane‐coated nanoparticles have been functionalized with lipid‐PEG‐anchored folate, which can be inserted postsynthesis to preserve the biological function of the natural coating (Figures 2a–2c).^10^ The resulting targeted nanoparticle showed significantly enhanced uptake in a folate receptor‐overexpressing KB cell line (a subline of the keratin‐forming tumor cell line HeLa). In a different work, doxorubicin‐loaded heparin‐folate‐paclitaxel nanoparticles were able to both extend release of the drug in vitro and localize within tumors in vivo, significantly slowing their growth (Figure 2d).^30^ Microbubbles have been of interest lately in ultrasound‐mediated cancer therapy, and folate‐targeted microbubbles have been fabricated, improving the ability of the particles to localize to tumor and subsequently ablate them.^31^ Beyond drug delivery, this targeting strategy has also been used extensively in imaging applications. For example, folate‐functionalized iron oxide nanoparticles have been used to detect cancer using magnetic resonance imaging (MRI).^32^ Functionalization with folate allowed the particles to be taken up preferentially by cancer cells while retaining their superparamagnetic properties. Other folate‐targeted MRI contrast particles include gadolinium‐loaded dendrimers^33^ and rare‐earth metal yttrium‐oxide particles that avoid the use of heavy metals for imaging.^34^

### 2.2 Riboflavin

Riboflavin, better known as vitamin B_2_, is a small molecule with potential implications for targeted delivery. The cofactors of riboflavin, flavin adenine dinucleotide (FAD) and flavin mononucleotide (FMN), are required for the metabolism of folate, and depletion of these cofactors has been found to slow metabolism within cells.^35^ The exact nature of riboflavin's role in cancer pathogenesis is unclear,^36^, ^37^ although it is known that riboflavin carrier protein is overexpressed on many types of cancer.^38^ This fact, along with the molecule's close relationship to folate metabolism, suggests that it can play a role in the design of cancer therapeutics and diagnostics. An FAD‐conjugated ultra‐small superparamagnetic iron oxide nanoparticle has been reported as a targeted imaging strategy for tumor vascular metabolism.^39^ The nanoparticles were able to effectively target riboflavin carrier protein and highlight sites of angiogenesis in vivo. A similar platform based on FMN as the targeting ligand has also been reported.^40^ For the delivery of cancer therapeutics, dendrimers have been used in conjunction with riboflavin as the targeting agent. Methotrexate‐riboflavin dendrimer conjugates were able to target and specifically inhibit the growth of KB cells in vitro.^41^ Despite the fact that the exact pathways and relationship between riboflavin and cancer have not been fully elucidated, the works here serve as a foundation for further study of the molecule as a targeting agent.

## 3 CARBOHYDRATES

### 3.1 Simple sugars

The targeting of simple carbohydrate receptors using naturally occurring sugars is a commonly used method of modulating nanoparticle localization. One such example is mannose, which is expressed on many types of cells, notably immune cells.^42^ Targeting of dendritic cells, which highly express mannose receptors, has been used to enhance the activity of nanoparticle vaccine formulations. In one example, lipid‐calcium‐phosphate (LCP) nanoparticles loaded with both an adjuvant, CpG oligodeoxynucleotide, and a melanoma‐associated antigen, Trp2, were functionalized with mannose to create a therapeutic anticancer vaccine.^43^ The nanoformulation was able to reduce tumor growth in both subcutaneous and metastatic models of murine B16‐F10 melanoma. In a follow‐up work, the LCPs were combined with liposome‐protamine‐hyaluronic acid nanoparticles delivering siRNA against TGF‐β as a combinatorial treatment, further boosting vaccine efficacy.^44^ Mannose can also be used as a ligand to target cancer through tumor‐associated macrophages, which aid the growth of tumors by releasing protein factors that stimulate tumor growth.^45^ A PEGylated nanoparticle platform has been reported that takes advantage of this targeting mechanism.^46^ Mannose‐modified particles are coated in a pH‐sensitive outer layer of PEG, which is sheddable in low pH tumor microenvironments. This results in exposure of a mannose‐functionalized layer that enables efficient binding to tumor‐associated macrophages and subsequent payload delivery.

**Figure 3:**
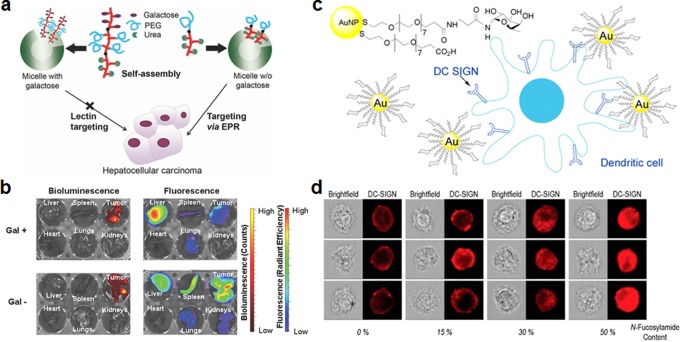
Examples of targeting strategies using carbohydrates. (a, b) Galactose‐functionalized micelles for liver targeting. (a) Schematic of micelles functionalized with and without galactose‐PEG ligands. Galactose‐targeted particles are localized in the liver, and nontargeted particles are localized in the tumor. (b) In vivo biodistribution of galactose‐targeted and nontargeted particles 48 hr after intravenous administration. Bioluminescence originates from luciferase‐expressing tumor cells. (c, d) Glycan‐functionalized gold nanoparticles (AuNPs) for dendritic cell targeting. (c) Glycomimetic α‐fucosylamide functionalization scheme of AuNPs, these nanoparticles can be taken up preferentially by dendritic cells via DC‐SIGN lectins. (d) Internalization of α‐fucosylamide functionalized AuNPs (with either 0, 15, 30, or 50% α‐fucosylamide linked ligands on the surface) by dendritic cells. Increasing the amount of α‐fucosylamide on the surface increases percent of nanoparticles internalized by the dendritic cells. (a, b) Adapted with permission from ref. 49. Copyright 2014 by John Wiley & Sons, Inc. (c, d) Adapted with permission from ref. 60. Copyright 2014 by American Chemical Society

Galactose is a simple sugar that is also frequently used for targeted delivery, and it has a high affinity for asialoglycoprotein receptors found on hepatocytes.^47^ As an example, PEG‐stabilized gold nanoparticles with galactose as a targeting ligand exhibited increased uptake in a HepG2 liver cell line.^48^ The targeting effect was shown to be specific, as the presence of free asialoglycoprotein receptor ligands markedly decreased gold nanoparticle uptake. Further, it has been demonstrated that galactose‐functionalized micelles can target the liver with high efficiency in vivo (Figures 3a and 3b).^49^ Galactose‐modified liposomes can be highly effective at hepatic siRNA delivery; compared with the bare nucleic acid, liposome‐encapsulating siRNA was delivered much more efficiently to the liver in vivo.^50^ Galactose‐mediated liver targeting has also been used for antiviral therapy, enabling delivery of antiviral p41 peptide nanocomplexes against hepatitis C.^51^ For imaging purposes, iron oxide nanoparticles functionalized with a galactose‐containing polymer displayed increased accumulation in the liver, demonstrating their utility as contrast agent for liver MRI.^52^

### 3.2 Glycans and glycan‐binding lectins

Glycans represent more complex carbohydrates that are often anchored onto either membrane lipids or proteins, and these biomacromolecules have important implications in many natural binding interactions.^53^, ^54^ They have been used to improve biocompatibility and stability of nanoparticles in protein‐rich solutions and to add lectin targeting abilities. In a recent work, N‐acetylglucosamine (GlcNAc) was used to modify gold nanorods.^55^ The modification reduced macrophage uptake in vitro and enabled interaction of the nanoparticles with carbohydrate‐binding proteins. Dendritic cell‐specific intercellular adhesion molecule‐3‐grabbing nonintegrin (DC‐SIGN) is a popular C‐type lectin on the surface of dendritic cells that can be targeted by highly mannosylated or Lewis‐type glycan structures.^56^ Both Lewis X and Lewis B glycans have been used to modify liposomes, and it was demonstrated that PEGylation inhibits their binding activity.^57^ The same modifications were later made on antigen‐carrying liposomes, and the targeted formulations led to markedly enhanced presentation of the antigens to CD4^+^ and CD8^+^ effector T cells driven by dendritic cells pulsed with the nanoformulation.^58^ In another example, Lewis X‐functionalized iron oxide nanoparticles were used as a means for both dendritic cell detection and isolation.^59^ Gold nanoparticles have also been used to show preferential targeting and internalization by targeting DC‐SIGN on dendritic cells (Figures 3c and 3d).^60^ Gangliosides such as GM1 are lipid‐anchored glycan structures that have been functionalized on nanoparticle surfaces for the binding and detection of toxins, including cholera toxin secreted by *Vibrio cholera*.^61^ The fact that such gangliosides can also mediate direct binding interactions with pathogens such as *Streptococcus pneumoniae* suggests that such ganglioside‐functionalized nanoparticles have utility for bacteria targeting applications.^62^, ^63^

Conversely, lectins, which are a class of proteins that regulate bioadhesion and cell recognition,^64^, ^65^ have been used as a means to target glycan structures. This has important implications for gastrointestinal tract targeting, as their bioadhesive properties can enable them to navigate through mucosal layers, cross epithelial barriers, and enter cells.^64^, ^66^ *Ulex europaeus* 1 lectin (UEA‐1) was shown to be taken up quickly and preferentially by M cells in vivo.^67^ Due to the role of M cells in immunity, a poly(lactic‐*co*‐glycolic acid) (PLGA) nanoparticle vaccine against hepatitis B was targeted to M cells using UEA‐1, and it was observed that the immune response was improved compared to a nontargeted control.^68^ A similar platform was reported using chitosan nanoparticles coated with a UEA‐1‐conjugated alginate gel capable of protecting the antigen from the acidic stomach environment,^69^ and a nasally administered vaccine against *Staphylococcus aureus* has also been developed using similar principles.^70^ Wheat germ agglutinin (WGA) from *Triticum vulgaris* is another lectin that has been used for targeted nanoparticle delivery. It has been shown that functionalization of PLGA nanoparticles with WGA can facilitate increased endocytic uptake.^71^ In a rat model, WGA nanoparticles improved the bioavailability of the steroid medication budesonide in the lungs when administered intratracheally compared with an unconjugated control.^72^ Regarding cancer therapy, WGA‐conjugated PLGA nanoparticles have been used to deliver the chemotherapeutic paclitaxel to different cell types and have shown promise as a mode of treatment.^73^, ^74^

## 4 PEPTIDES

### 4.1 Targeting peptides

Peptides are a popular class of targeting ligand, and can be used to help modulate bodily localization.^75^, ^76^ While many peptides that are identified via the phage display screening approach result in novel sequences, there are many examples of ligands that are either naturally occurring or derived from naturally occurring proteins. A prime example is arginylglycylaspartic acid (RGD), a sequence motif identified in fibronectin that binds cell surface receptors known as integrins.^77^ α_v_β_3_ is an important RGD‐binding integrin implicated in tumor angiogenesis and has served as the target for numerous RGD‐functionalized nanotherapeutics.^78^ Given its utility as a cancer‐targeting agent, RGD has been widely employed to create targeted therapeutic and imaging platforms.^79^, ^80^ RGD‐functionalized mesoporous silica‐encapsulated gold nanoparticles loaded with DOX were used as a combination photothermal therapy and chemotherapy to promote significant control of tumor growth while lowering systemic toxicity.^81^ In an example of gene silencing, RGD‐decorated chitosan nanoparticles loaded with siRNA were examined for their ability to selectively deliver their cargo to tumor cells, and the strategy promoted antitumor effects in a model of ovarian carcinoma.^82^ RGD is also a highly utilized peptide for targeted imaging and detection techniques. In one example, quantum dots coated with paramagnetic lipids and RGD‐conjugated lipids were tested as bimodal imaging probes.^83^ By further modifying nanoparticles with additional binding peptides, researchers were able to target both integrin α_IIb_β_3_ and P‐selectin on activated platelets using RGD and EWVDV peptides, respectively, resulting in better retention under flow conditions.^84^ A recently reported novel application of natural peptide functionalized nanoparticles involves their use as hemostatic agents.^85^ Flexible nanoparticles were conjugated with both an RGD‐containing peptide sequence as well as a von Willebrand factor‐binding peptide derived from factor VIII, helping to promote platelet aggregation and reduce bleeding time in a mouse model.

**Figure 4:**
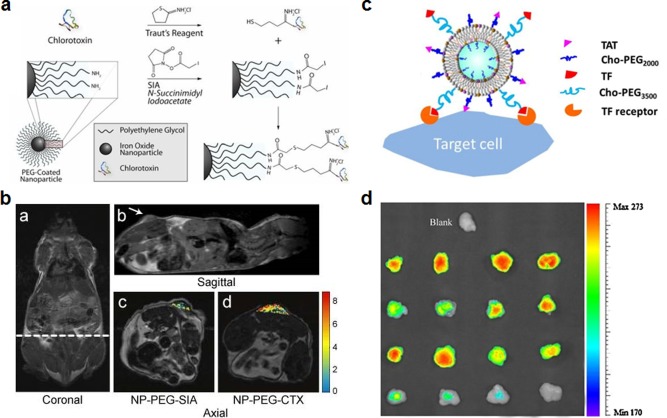
Examples of targeting strategies using peptides. (a, b) Chlorotoxin (CTX)‐functionalized iron oxide nanoparticles for glioma targeting. (a) Schematic showing conjugation scheme for CTX functionalization of PEGylated iron oxide nanoparticles. (b) MRI images of CTX nanoprobe localization in tumor xenograft. CTX‐targeted nanoparticles showed higher accumulation in tumors than a control nanoparticle, NP‐PEG‐SIA. (c, d) TAT‐functionalized liposomes for enhanced tumor entry. (c) Schematic of nanoformulation functionalized with both TAT peptide and transferrin (TF) using cholesterol‐PEG (Cho‐PEG) conjugates. TF enables cell targeting while the TAT enhances cellular entry. (d) Fluorescently labeled liposome uptake by tumors in an in vivo xenograft model. Formulations, from top to bottom, were liposomes with TAT and TF, liposomes with TAT only, liposomes with TF only, and bare liposomes. (a, b) Adapted with permission from ref. 92. Copyright 2008 by John Wiley & Sons, Inc. (c, d) Adapted with permission from ref. 107. Copyright 2013 by Elsevier

Toxins are crafted by nature to target cellular surfaces, and their activity on host cells can cause major disruption of cellular processes.^86^ Some toxins have binding sequences that are designed to target membrane receptors with high specificity. For example, chlorotoxin is a peptide from scorpion venom that was originally found to block chloride ion channels, and is a highly used targeting peptide.^87^ Although the exact mechanism of chlorotoxin's binding is still under some controversy,^88^ it has been shown to preferentially bind several types of cancers.^89^, ^90^ Gliomas in particular are highly sensitive to the peptide, which binds to the cells via both high and low affinity sites,^91^ making it a good ligand to use for imaging of brain tumors.^92^, ^93^, ^94^ Multiple platforms have used chlorotoxin to target particles to cancer cells for both imaging and therapeutics.^91^, ^92^, ^93^ Targeting of nanoparticles to the brain was greatly enhanced by the addition of chlorotoxin to the surface of iron oxide particles (Figures 4a and 4b).^92^ Chlorotoxin has been used to “paint” tumors using fluorescent dyes as well,^94^ and other imaging modalities, including quantum dots, have been used in conjunction with this targeting method.^95^ In other cancer types such as metastatic breast cancer, chlorotoxin was able to enhance the cellular uptake and cytotoxicity of liposomes in a murine model of the disease.^96^ Other than its specificity to cancer, chlorotoxin has shown efficacy in targeting Parkinson's disease (PD) as well, as it has a specificity for proliferating endothelial cells.^97^ This allowed researchers to target brain microvascular endothelial cells and deliver levodopa within the brain to treat the disease.^98^

Candoxin is a component of snake venom from *Bungarus candidus* that produces a neuromuscular blockade in the central nervous system and was recently shown to have brain targeting properties.^99^ The toxin binds to nicotinic acetylcholine receptors, which facilitates their uptake into brain cells.^100^ Researchers have taken advantage of the toxin's binding specificity to derive a peptide sequence (CDX) for use in brain‐targeted nanoparticle delivery.^101^ PEG‐poly(lactic acid) (PEG‐PLA) micelles coated with CDX were able to achieve a significant increase in brain distribution, highlighting their potential use as a targeting mechanism for human glioblastoma multiforme therapies. The same group later used a more stable D‐peptide version of the CDX ligand to target liposomal doxorubicin in a mouse model of glioma, demonstrating enhanced brain uptake and increased survival with the targeted formulation.^102^ To further increase efficacy, liposomes were later functionalized with both CDX and an RGD‐containing peptide, and the resulting nanoformulation showed better tumor control compared with liposomes functionalized with either ligand individually.^103^

### 4.2 Cell penetrating peptides

Rather than enabling targeting to a specific bodily location, cell penetrating peptides are natural moieties often employed by pathogens that aid in the disruption of cell membrane bilayers and allow entry into a cell.^104^, ^105^ Researchers have employed such peptides to improve intracellular delivery of nanoparticle payloads. HIV trans‐activator of transcription (TAT) is a protein with cell‐penetrating properties that the virus uses to efficiently enter cells via endocytic pathways.^106^ A TAT‐derived peptide has been used frequently as a surface ligand for cancer drug delivery to increase uptake of chemotherapeutic payloads. Liposomes functionalized with both TAT and transferrin showed highly cancer‐specific delivery of cargo directly into tumor cells (Figures 4c and 4d).^107^ Tumors subject to nanoparticles functionalized with both TAT and transferrin had the highest intensity of fluorescent localization. A TAT‐functionalized PEG‐PLA platform coated in poly(methacryloyl sulfadimethoxine) (PSD)‐b‐PEG has been used as a responsive targeted drug delivery system.^108^ The particles exhibited increased uptake at lower pH values due to exposure of the TAT ligands after shedding of the PSD‐b‐PEG layer. In addition to potential drug delivery applications, TAT has been tested for gene therapy uses. TAT‐functionalized liposomes were reported to induce transfection in lung carcinoma cells,^109^ and TAT‐conjugated PEG‐polyethylenimine nanoparticles were successfully used for in vivo gene delivery to the lungs.^110^ For imaging purposes, a TAT‐functionalized dual fluorochrome magnetic particle has been reported for looking at the metabolism and movement of cells in vivo using both MRI and fluorescent imaging techniques.^111^ The TAT peptide may also be helpful for brain delivery, where PEG micelles functionalized with the peptide and loaded with the antibiotic ciprofloxacin have the potential to treat brain infection.^112^ These TAT peptide‐functionalized micelles showed significant brain uptake in rats 4 hr after intravenous injection. PEGylated chitosan nanoparticles with TAT have also been shown to enter the brain after intranasal administration, and these particles have potential in the treatment of neurodegenerative disorders.^113^

Penetratin is a cell‐penetrating peptide derived from *Drosophila* Antennapedia homeodomain that also aids in delivery across the cell membrane.^114^ Gold nanoparticles with penetratin on their surface fabricated using laser ablation in a solution containing the peptide were able to enter cells more effectively than their nonfunctionalized counterparts.^115^ Curcumin, used against neurodegenerative disorders, was delivered to the brain using penetratin‐functionalized micelles.^116^ Fluorescent‐magnetic nanoparticles have also been coated with the peptide, and the resulting targeted formulation exhibited enhanced endocytosis in the presence of a magnetic field.^117^ Penetratin, like TAT, has shown benefit as a brain delivery platform, where functionalized PEG‐PLA nanoparticles were able to more efficiently cross the blood‐brain barrier (BBB) in vivo when compared with non‐penetratin decorated nanoparticles.^118^

## 5 PROTEINS

### 5.1 Transferrin family proteins

Transferrins are glycoproteins which bind iron ions in the serum and transport them to cells via transferrin receptors.^119^, ^120^ These receptors can be highly overexpressed on certain cancers, and transferrin receptor‐targeting strategies represent one of the widely studied methods for targeted delivery of therapeutic and imaging agents.^120^ Transferrin functionalization has been reported for many different nanoparticle platforms, including liposomes,^121^, ^122^, ^123^ polymeric nanoparticles (PNPs),^124^, ^125^ gold nanoparticles,^126^, ^127^ and iron oxide nanoparticles.^127^ Interestingly, it was found in one study that functionalizing gold nanoparticles with transferrin did not significantly influence their overall biodistribution, but it did strongly influence localization to certain cell types within different organs and within tumors.^126^ Environmentally responsive microgels decorated with transferrin have been designed for cancer treatment and displayed triggered release of a doxorubicin payload in the low pH of lysosomes.^128^ This targeting strategy has also been used for photothermal therapy, where gold nanorods targeted to the transferrin receptor showed significant excitation upon exposure to a low energy laser due to enhanced cellular uptake.^129^ Cadmium sulfide quantum dots with transferrin on the surface have been designed for imaging purposes, and the particles were readily taken up by cancer cells, resulting in bright illumination.^130^

**Figure 5:**
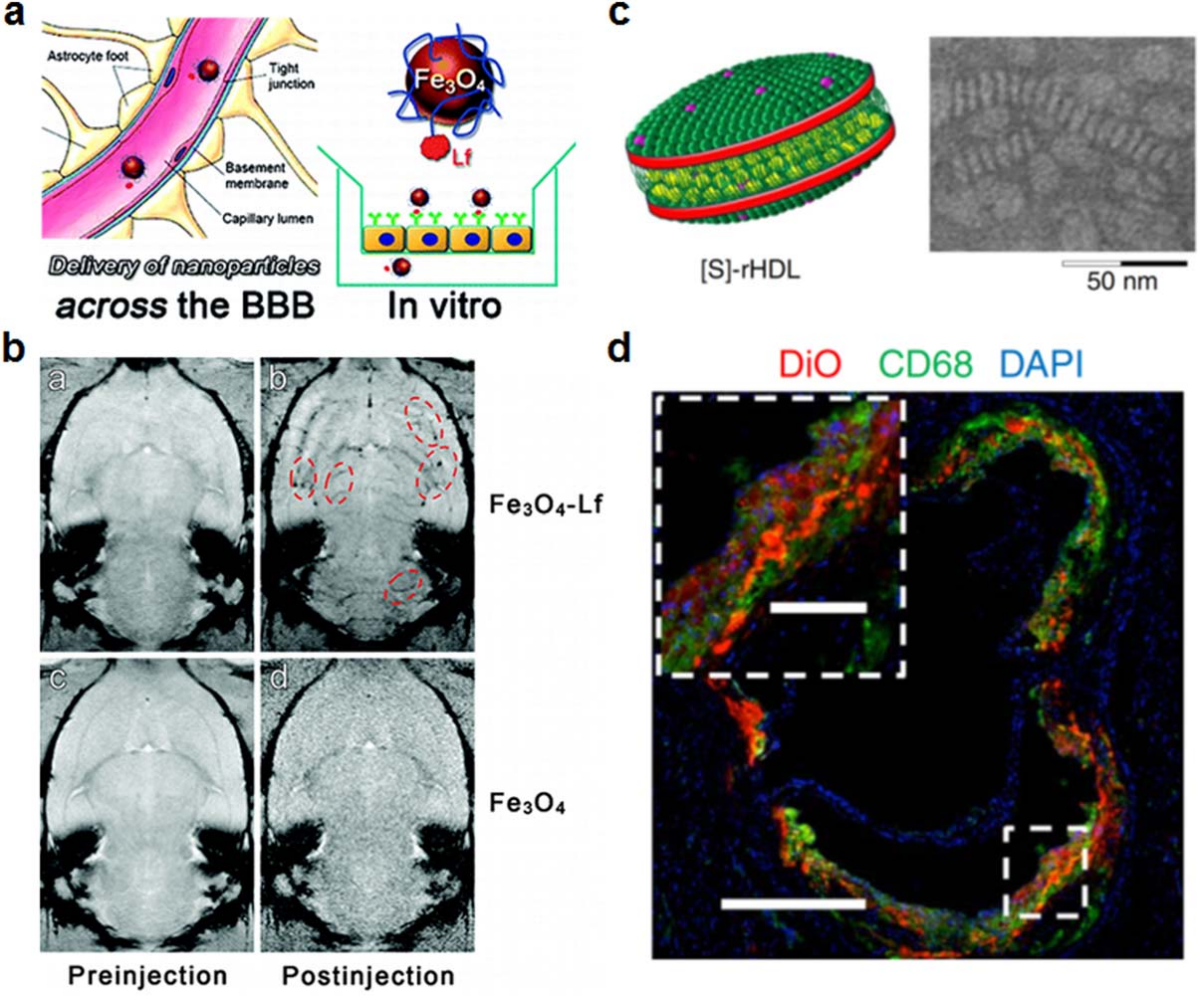
Examples of targeting strategies using proteins. (a, b) Lactoferrin (Lf)‐functionalized iron oxide nanoparticles for delivery across the BBB. (a) Schematic of Lf‐coated iron oxide nanoparticles demonstrating the interactions between the nanoparticles and brain endothelial cells. (b) In vivo MRI image showing brain uptake of Lf‐coated iron oxide nanoparticles. Red dotted lines encircle areas of contrast where nanoparticles are present. (c, d) HDL nanoparticles for delivery to atherosclerotic plaque. (c) Schematic (left) and transmission electron microscopy (TEM) image (right) of statin‐loaded recombinant HDL nanoparticles ([S]‐rHDL). TEM image shows [S]‐rHDL were 26 nm in diameter. (d) Fluorescent imaging shows colocalization between the [S]‐rHDL particles labeled with dye and macrophages (CD68) at sites of plaque in an aorta. Scale bars = 400 μm for main panel and 100 μm for inset. (a, b) Adapted with permission from ref. 135. Copyright 2012 by American Chemical Society. (c, d) Adapted with permission from ref. 147. Copyright 2014 by Macmillan Publishers Ltd.

Lactoferrin is another glycoprotein in the transferrin family that is naturally produced in milk, saliva, and tears. It plays a role in transfer of immunity to infants, immune response, and protection against microbial infection.^131^, ^132^ Lactoferrin has been used as a natural brain‐targeting ligand due to its receptor‐mediated ability to cross the BBB, one of the greatest challenges in the treatment of brain cancers.^133^ Along these lines, PEG‐coated bovine serum albumin nanoparticles functionalized with lactoferrin have been used to deliver doxorubicin for glioma treatment in vivo.^134^ The targeted particles were able to increase the efficacy of doxorubicin against gliomas and increase the nanoparticle concentration in tumors compared to nontargeted particles. Lactoferrin has also been used to improve brain imaging techniques; coated super‐paramagnetic iron oxide nanoparticles were used to enhance imaging resolution for brain tumor detection (Figures 5a and 5b).^135^, ^136^ Nanoparticles coated with lactoferrin and labeled with a far‐red fluorescent dye have also been evaluated for brain tumor imaging and showed promising results for this application.^137^ In addition to cancers, brain targeting via lactoferrin can also be useful for treating neural disorders such as Alzheimer's. Lactoferrin‐coated nanoparticles delivering deferasirox, an iron chelator, were able to protect the brain from amyloid accumulation and subsequent neurodegeneration in a rat model of the disease.^138^ For regulating the chemical balance within the brain, lactoferrin‐functionalized liposomes were used to deliver senktide, an NK_3_ receptor agonist that normally does not cross the BBB but is important in the evaluation of novel antipsychotics.^139^

### 5.2 Lipoproteins

High‐density lipoproteins (HDL) are involved in the transportation of lipid material and have implications in many common diseases, including heart disease and cancer.^140^, ^141^ Due to its ability to target specific cell types, HDL particles have been used extensively in drug delivery. Reconstituted HDL nanoparticles containing fluorescent calcium carbonate were able to serve as a probe for lung cancer detection in vivo.^142^ Their targeting mechanism relied on targeting scavenger receptor class B member I (SR‐BI), which is highly expressed on some cancerous cells and has an affinity to HDL.^142^ In another example, lymphoma cells that express SR‐BI were also targeted effectively in vivo with synthetic, gold‐templated HDL nanoparticles.^143^ Given that the main role of HDL is lipid transport, it also plays a pivotal role in atherosclerosis.^144^, ^145^ HDL‐mimetic particles have been used to target atherosclerotic plaques for imaging,^146^ and HDL has also been used to deliver therapeutic statins to reduce inflammation and atherosclerotic plaque buildup (Figures 5c and 5d).^147^

### 5.3 Adhesion proteins

Cell adhesion molecules are proteins that cells use to bind other cells or extracellular matrix, and these comprise of immunoglobulins, cadherins, selectins, and integrins as the main subtypes.^148^, ^149^, ^150^ They are employed by a variety of cells, most notably on blood components, such as platelets and leukocytes, or those comprising endothelial surfaces. Despite their favorable binding characteristics and often site‐specific upregulation, the use of adhesion proteins in their entirety has not been commonly reported, most likely due to the difficulty associated with functionalizing nanoparticles using membrane‐bound proteins. In one example, P‐selectin glycoprotein ligand‐1 conjugated onto microbeads was used to study neutrophil rolling interactions as well as elucidate binding interactions of the ligand with E‐selectin.^151^, ^152^ To overcome difficulties associated with their use, researchers often rely on the use of binding domains derived from these adhesion proteins, which enable more facile conjugation to nanoparticle surfaces. Leukocyte‐mimetic iron oxide nanoparticles were created by coating with lymphocyte function‐associated antigen 1 I domain, which targets intercellular adhesion molecule 1 (ICAM‐1).^153^ These particles were able to target inflamed tumor vasculature due to leukocyte‐endothelial cell interactions under inflammatory conditions, and could serve as a cancer imaging agent. Flexible particles coated in the GPIbα amino terminal domain, a derivative of a critical binding protein on platelets, were able to partly mimic the binding properties of the cells by showing affinity for von Willebrand factor.^154^

## 6 PATHOGEN‐DERIVED PARTICLES

### 6.1 Viruses

**Figure 6:**
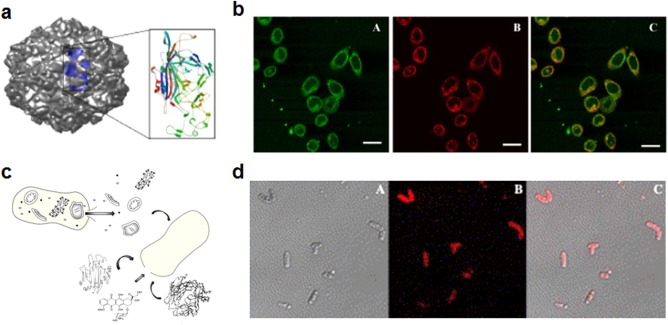
Examples of targeting strategies using pathogen‐derived particles. (a, b) Canine parvovirus‐like particles (CVP‐VLPs) for delivery to tumors. (a) Model of the protein capsid with a single subunit depicted on the inset. (b) CVP‐VLPs were internalized by HeLa cells, as shown by colocalization of antibody‐labeled CVP‐VLPs (green) and dye‐conjugated transferrin (red). Scale bar = 25 µm. (c, d) Bacteria ghosts for delivery to tumors. (c) Schematic showing formation of bacterial ghosts through evacuation of the intrabacterial contents and loading with therapeutic or imaging agents. (d) Doxorubicin‐loaded bacterial ghosts as targeted drug delivery vehicles. Microscopy images show bacterial ghosts (bright field) and doxorubicin (red) overlaid. (a, b) Adapted with permission from ref. 163. Copyright 2006 by BioMed Central Ltd. (c) Adapted with permission from ref. 168. Copyright 2014 by Medknow. (d) Adapted with permission from ref. 166. Copyright 2009 by Elsevier

Virus‐like particles (VLPs), mimicking naturally occurring viral capsids, have been studied for their ability to deliver different payloads.^155^, ^156^ These represent a promising form of targeted delivery given that many viruses gain cellular entry via specific interactions with cell surface receptors.^157^, ^158^ One example is the human rhinovirus, which targets the ICAM‐1 protein that is present on the surface of many immune cells.^159^ Hepatitis B envelope L proteins can form hollow nanoparticles with liver‐targeting properties, and this was leveraged to deliver human clotting factor IX genes for potential treatment of hemophilia B.^160^ A VLP based on the penton capsid proteins of adenovirus serotype 5 was likewise able to recapitulate the cellular targeting and entry characteristics of the original virus.^161^ Derivatives of this platform have been used to deliver doxorubicin to HER2‐expressing cells for use in cancer therapy.^162^ Canine parvovirus capsid‐based VLPs were shown to target transferrin receptor‐overexpressing cells, and the effect was demonstrated using fluorescent dye‐conjugated particles as a proof‐of‐concept (Figures 6a and 6b).^163^ Interestingly, VLPs have also been used to encapsulate other nanoparticles for enhanced imaging techniques. Hepatitis B VLPs were loaded with iron oxide nanoparticles, and the resulting formulation could be efficiently taken up intracellularly for improved MRI contrast.^164^

### 6.2 Bacteria

Like viruses, bacteria have also evolved high affinities to specific mammalian cells and tissues. Many bacteria display ligands that specifically target cellular receptors, an example being SraP on *Staphylococcus aureus*, which specifically binds platelets.^165^ Some bacteria actually have natural cancer‐targeting properties due to adhesion molecules on their surfaces. For example, bacterial ghosts derived from *Mannheimia haemolytica* bacteria were loaded with doxorubicin and demonstrated the ability to target colorectal adenocarcinoma cells more specifically than control ghosts derived from *Escherichia coli* (Figures 6c and 6d).^166^ Due to their affinity to certain immune cells, which are adept at recognizing and binding bacteria, bacterial ghosts have also been used to improve vaccine design.^167^, ^168^ Likewise, bacterial outer membrane vesicles (OMVs), which have been recognized as promising delivery vehicles, have been used to deliver antigenic material while also boosting immunogenicity.^169^ For example, *E. coli* OMVs have been used to generate strong immune responses against green fluorescent protein, and the resulting titers were much higher than what could be achieved by administration of the free protein alone.^170^ There are many future applications that can be pursued given the wide range of specificities that bacteria demonstrate, including endothelial cells and epithelial cells.^168^, ^171^ Additionally, it has been demonstrated that bacterial membrane can be coated onto nanoparticle surfaces, further introducing new functionalities to these naturally derived platforms.^172^

## 7 MAMMALIAN CELL MEMBRANES

### 7.1 Exosomes

**Figure 7:**
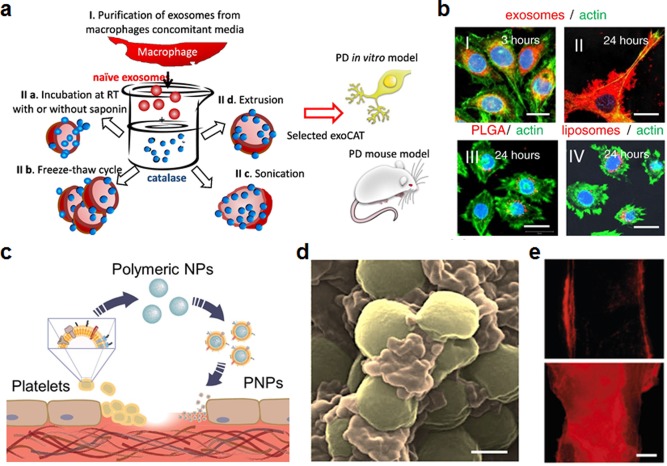
Examples of targeting strategies using mammalian cell membranes. (a, b) Macrophage exosomes for delivery to the brain. (a) Schematic of macrophage exosome derivation and application. Catalase‐loaded macrophage exosomes were designed as a brain‐targeted Parkinson's disease (PD) therapy. (b) Exosome formulations were tested for uptake by PC12 neuronal cells compared with control particles. Macrophage‐derived exosomes (I and II) showed greatly enhanced uptake compared to nontargeted polymeric PLGA particles (III) and liposomes (IV) at 24 hr. Scale bar = 20 μm. (c–e) Platelet membrane‐coated nanoparticles (PNPs) for delivery to bacteria and damaged vasculature. (c) Schematic of fabrication and targeting abilities of PNPs. (d) Pseudocolored scanning electron microscopy (SEM) image of PNPs (orange) naturally targeting MRSA252 bacteria (gold). Scale bar = 400 nm. (e) Targeting of fluorescently labeled PNPs (red) to intact carotid artery (top) and damaged artery (bottom). PNPs show preferential binding to damaged areas. Scale bar = 500 μm. (a, b) Adapted with permission from ref. 180. Copyright 2015 by Elsevier. (c–e) Adapted with permission from ref. 189. Copyright 2015 by Macmillan Publishers Ltd.

The direct use of naturally derived cell membranes in nanomedicine is a growing field that has the potential to lead to novel and improved delivery platforms.^17^, ^173^, ^174^ One major advantage of the approach is that it enables direct use of multivalent cell membrane markers concurrently. This can include both targeting molecules as well as immunomodulatory surface markers, which better enable platforms developed using this approach to excel within complex, biological systems. One type of natural membrane structure that has been employed is exosomes, which are vesicular fragments filled with intracellular content that break off of cells and play roles in signaling and transport.^175^ They have a natural affinity to some locations within the body due to their role in signaling and have wide‐ranging implications for drug delivery and imaging applications. Recently, the natural affinity of tumor‐derived exosomes to specific organs was studied.^176^ It was shown that, depending on the source tumor, the exosomes had varied biodistributions; this correlated with sites of future metastasis and was integrin‐dependent. In a different study, macrophage‐derived exosomes were used for delivery of doxorubicin due to their natural targeting ability to tumors.^177^ It was shown that the tumor‐targeting mechanism of the exosomes could be attributed to several CAM‐like and other adhesion molecules on their surfaces. Exosomes from dendritic cells can be used to modulate the immune system by interacting with the T cells responsible for downstream immune activity.^178^ When using exosomes from immature dendritic cells pretreated with interleukin‐10, it has been possible to generate immune tolerance for alleviating arthritic conditions.^179^ Additionally, exosomes derived from macrophages can deliver cargo to the brain through receptor‐based interactions with potential implications for PD therapy (Figures 7a and 7b).^180^

### 7.2 Platelets

Platelets are critical in maintaining hemostasis and are the front line in wound healing, where they mediate the clotting cascade.^181^, ^182^ Due to their complex nature, including the fact that their membrane displays numerous cell and extracellular matrix adhesion proteins, platelets are implicated in many pathologies, including cancer,^183^ cardiovascular disease,^184^ and infections.^185^ Cell membrane‐coated nanoparticles have recently been reported that mimic the natural function of the source cells.^21^ The faithful translocation of membrane onto the nanoparticle surface preserves the original functionality, including the ability to actively reduce immune uptake^186^ or to bind cell‐specific toxins.^187^, ^188^ Along these lines, a platelet membrane‐coated nanoparticle (PNP) has recently been reported that takes advantage of the varied binding properties of platelets (Figures 7c–7e).^189^ The nanoparticles, which consist of PLGA as the core material and a naturally derived platelet membrane bilayer as the shell, recapitulate many of the original binding properties of platelets. It was shown that the PNPs could be used for multiple purposes, including for the targeting and treatment of antibiotic‐resistant bacteria as well as for delivering cytotoxic payloads to regions of damaged vasculature for preventing restenosis.

Such membrane‐coated nanoparticles have also been used for cancer treatment, where platelets and growing tumors have a very multifaceted and complex relationship.^183^ There are several methods by which cancer cells can bind to and activate platelets, and platelet‐mimicking imaging and therapeutic platforms can use this to their advantage. Platelet membrane‐coated particles have recently been investigated for use as drug delivery vehicles to both tumors and circulating tumor cells.^190^ The nanoformulation was both loaded with doxorubicin and conjugated with tumor necrosis factor‐related apoptosis inducing ligand (TRAIL); they efficiently localized to tumor cells in vivo, resulting in impressive control of both tumor growth and the amount of metastatic nodules in the lung. In a similar example, silica particles functionalized with platelet membrane and conjugated with TRAIL were also able to reduce metastasis in a mouse breast cancer model.^191^

### 7.3 Leukocytes

White blood cells, or leukocytes, are immune cells that play a major role in the detection and elimination of foreign or malignant entities. Of note, leukocytes can target endothelium, and silica particles coated with membrane derived from these cells were shown to bind to endothelial cells in an ICAM‐1‐dependent manner.^192^ These membrane‐coated particles were able to successfully traverse the endothelium and retained their properties when administered in vivo. In a different example, silica particles coated with macrophage cell membrane were loaded with doxorubicin and used for tumor treatment.^193^ The particles exhibited extended blood circulation times and enhanced tumor accumulation, leading to significant control of tumor growth in a mouse xenograft model. Leukocyte membrane‐coated polymeric capsules were also shown to enhance cancer cell‐targeting capability compared with unmodified particles.^194^ In a recent study using Janus particles in which one side was coated with gold and the other side with leukocyte membrane, it was demonstrated that the particles with membrane coatings could preferentially bind to cancer cells over healthy endothelial cells.^195^ Interestingly, the authors noted that the particles entered via the membrane‐coated hemisphere, demonstrating the utility of the biological coating for cancer targeting.

### 7.4 Stem cells

Mesenchymal stem cells (MSCs) have unique tumor‐targeting abilities given that they are recruited by tumors to enhance their proliferation.^196^, ^197^ These are mediated in part by direct cell‐cell interactions, suggesting utility of stem cell‐derived membrane for targeted delivery.^198^ “Nanoghosts” derived directly from MSC membranes were recently reported as a potential platform for targeted therapy of prostate cancer.^199^ The vesicles were loaded with soluble TRAIL (sTRAIL) by a physical extrusion method and were shown to retain their tumor‐binding properties both in vitro and in vivo. Compared with free sTRAIL, the nanoghost formulation showed significantly enhanced control of tumor growth. Further, the particles were shown to be nonimmunogenic, highlighting their potential for translation. Iron oxide nanoparticles coated with stem cell membrane have also been recently reported that have potential for both therapeutic and diagnostic applications.^200^ The coating process involved a sonication method that forced cell membrane to reassemble onto the surface of the nanoparticles, and the final particles were shown to exhibit reduced macrophage uptake compared with bare particles.

### 7.5 Cancer cells

Cancer cells often upregulate a variety of integrins and receptors on their surfaces that allow them to spread, bind, and aggregate together to metastasize and form tumors.^201^, ^202^, ^203^ While some of the binding mechanisms are heterotypic, homotypic binding mechanisms, where cancer cells bind to each other, have also been enumerated.^204^, ^205^ Recently, a cancer cell membrane‐coated nanoparticle (CCNP) has been fabricated to demonstrate the utility of a homotypic approach for cancer targeting.^206^ It was demonstrated that the particles retained membrane‐bound antigens that were present on the original cells. Compared with RBC membrane‐coated nanoparticles, the CCNPs bound preferentially to the original cancer cell. Further, it was shown that the CCNPs did not bind to normal, healthy cells, demonstrating the specificity of the targeting. While such a strategy needs to be further explored and validated, it represents a unique method for the fabrication of naturally cancer‐targeting nanoparticles.

## 8 CONCLUSIONS

Biomimetically targeted nanoparticle platforms leverage nature's own binding specificities to preferentially localize to regions of interest within the body. Unlike conventional targeting strategies, which often depend on approaches involving lengthy discovery and validation, the use of naturally occurring binding mechanisms has the potential to speed up workflows for new targeting ligand identification. Binding interactions among cells, small molecules, biomolecules, and surfaces are a fundamental part of biological systems, and the character of these interactions can vary greatly depending on the biological function. As illustrated in this review, natural biomimetic targeting strategies can be employed using anything from small molecule vitamins to entire cell membranes. Having a plethora of potential choices of targeting strategies for nanoparticle delivery is important, as each may be best suited for a different application. Selecting the correct approach will require consideration of many factors such as size and shape requirements, surface chemistry, intended fate, and route of administration.

Looking toward the future, it can be envisioned that multiligand strategies for targeted nanoparticle delivery will be increasingly explored. This stems from the fact that biological interactions are incredibly complex and seldom singular. By employing multiple targeting ligands, it may ultimately be possible to design delivery platforms with exquisite specificity and sensitivity. To accomplish this, it may be necessary to develop novel synthetic strategies for the controlled incorporation of multiple moieties onto the surface of nanoparticles. An alternative approach already described is the use of cell membrane coatings, a top‐down strategy that circumvents the need for complicated chemistries. Employing cell membranes not only enables the facile incorporation of multiple ligands and functionalities, but it also does so at stoichiometries found inherently in nature. Regardless of the method, as targeting strategies continue to develop and become more sophisticated over time, the ability of nanoparticle platforms to address pressing health issues will continue to improve.
